# Silkworm Pupa Protein Hydrolysate Induces Mitochondria-Dependent Apoptosis and S Phase Cell Cycle Arrest in Human Gastric Cancer SGC-7901 Cells

**DOI:** 10.3390/ijms19041013

**Published:** 2018-03-28

**Authors:** Xiaotong Li, Hongqing Xie, Yajie Chen, Mingzi Lang, Yuyin Chen, Liangen Shi

**Affiliations:** College of Animal Sciences, Zhejiang University, Hangzhou 310058, China; lixiaotong@zju.edu.cn (X.L.); xiehongqing@zju.edu.cn (H.X.); yajiesw@126.com (Ya.C.); 21617032@zju.edu.cn (M.L.); chenyy@zju.edu.cn (Yu.C.)

**Keywords:** antitumor activity, intrinsic apoptosis, cell cycle arrest, ROS, mitochondrial dysfunction, transcriptome analysis, silkworm pupae

## Abstract

Silkworm pupae (*Bombyx mori*) are a high-protein nutrition source consumed in China since more than 2 thousand years ago. Recent studies revealed that silkworm pupae have therapeutic benefits to treat many diseases. However, the ability of the compounds of silkworm pupae to inhibit tumourigenesis remains to be elucidated. Here, we separated the protein of silkworm pupae and performed alcalase hydrolysis. Silkworm pupa protein hydrolysate (SPPH) can specifically inhibit the proliferation and provoke abnormal morphologic features of human gastric cancer cells SGC-7901 in a dose- and time-dependent manner. Moreover, flow cytometry indicated that SPPH can induce apoptosis and arrest the cell-cycle in S phase. Furthermore, SPPH was shown to provoke accumulation of reactive oxygen species (ROS) and depolarization of mitochondrial membrane potential. Western blotting analysis indicated that SPPH inhibited Bcl-2 expression and promoted Bax expression, and subsequently induced apoptosis-inducing factor and cytochrome C release, which led to the activation of initiator caspase-9 and executioner caspase-3, cleavage of poly (ADP-ribose) polymerase (PARP), eventually caused cell apoptosis. Moreover, SPPH-induced S-phase arrest was mediated by up-regulating the expression of E2F1 and down-regulating those of cyclin E, CDK2 and cyclin A2. Transcriptome sequencing and gene set enrichment analysis (GSEA) also revealed that SPPH treatment could affect gene expression and pathway regulation related to tumourigenesis, apoptosis and cell cycle. In summary, our results suggest that SPPH could specifically suppress cell growth of SGC-7901 through an intrinsic apoptotic pathway, ROS accumulation and cell cycle arrest, and silkworm pupae have a potential to become a source of anticancer agents in the future.

## 1. Introduction

Silkworm, *Bombyx mori*, is an economic insect, which has been reared in China to produce silk since five thousand years ago. Silkworm pupae are the transitional form of larvae to adults, which contain abundant nutrients and bioactive substances. The protein content of fresh silkworm pupae is up to 60% after degreasing treatment, which contains substantial essential amino acids. The amino acid composition pattern of silkworm pupae obviously precedes the ideal amino acid pattern recommended by the Food and Agriculture Organization and the World Health Organization, with 36% amino acid composition [1,2]. Moreover, fat occupies about 25–30% weight of the silkworm pupae, and large amounts of oil have been extracted from silkworm pupae to produce industrial products in many countries [3]. After extractions of protein and oil, the remainder compounds of silkworm pupae contain about 36% chitin, which can be a reliable source of chitosan in the food industry [4]. Therefore, silkworm pupae are a promising source of biological resources.

During long-term domestication, the high nutrient value and health protective effects of silkworm pupae were gradually determined and subsequently utilized. Silkworm pupa powder intake can significantly promote fat metabolism and exert neuroprotective function by decreasing oxidative stress [5,6]. Recent studies on silkworm pupae mainly focused on the functions of bioactive peptides obtained by enzymatic hydrolysis. Angiotensin-I-converting enzyme (ACE) is a crucial enzyme in blood pressure regulation. Several ACE inhibitory peptides were accessed by performing protein hydrolyzing of silkworm pupae, and the bioactive peptides of silkworm pupae have been suggested to be effective in anti-hypertension [7,8,9]. Zhang et al. indicated the inhibitory effects of peptides from silkworm pupae on α-glucosidase trapping and suggested the potential use of silkworm pupae to treat diabetes [4]. In addition, enzymatic hydrolysates of silkworm pupae can prohibit the differentiation of preadipocytes and adipogenesis, which could be used to treat dietary obesity in the future [10].

Cancer is a noticeable public health problem worldwide and causes thousands of deaths annually. Cancer treatments largely depend on surgical operation, chemotherapy and radiotherapy, which usually lead to massive cell killing without specificity and cause many serious side effects on normal cells. Therefore, novel tumour-oriented drugs that could specifically kill cancer cells effectively with minimal toxicity to healthy cells must be developed. Compared with other anticancer agents, antitumour bioactive proteins and peptides have many advantages, such as excellent cell diffusion and permeability and specificity to certain tumourigenesis signal pathways without genotoxic effects [11]. Insect-derived antitumour peptides have been identified, and their antitumour mechanisms have been investigated in the past years [12]. Pierisin-1, an antitumour peptide purified from the pupae of *Pieris* species, can induce cytotoxicity and apoptosis of many human cancer cell lines with ADP-ribosylation activity [13]. Subsequently, alloferons were characterized from the blood of *Calliphora vicina* and exhibited stimulation of natural immunocyte activity and interferon synthesis and displayed antiviral and antitumour efficiency in mice [14]. In addition, a pentapeptide with amidated C-terminus was identified in wild silk moth *Antheraea yamammai* larvae and can suppress rat hepatoma cell proliferation by inducing cell-cycle arrest [15]. Melittin, a peptide derived from bee venom, can induce apoptosis of human hepatocellular carcinoma by activating the TAK1-JNK/p38 pathway and inhibiting the IκBα kinase-NFκB pathway [16]. The cecropin peptide of *Musca domestica* can exert cytotoxicity to BEL-7402 hepatocellular carcinoma cells in vivo without side effects [17]. Although anticancer peptides that originated from insect have been identified constantly, antitumour effect of peptides or protein hydrolysates that derived from silkworm are rarely discovered.

To determine the antitumour effect of the components of silkworm pupa, we performed enzymatic hydrolysis of defatted silkworm chrysalis using Alcalase. The cytotoxicity of silkworm pupa protein hydrolysate (SPPH) was then examined in seven different cancer cells, and we found that SPPH can specifically inhibit the growth of SGC-7901 cells in a dose- and time-dependent manner. Changes in morphology were also detected. Flow cytometry assays implied that SPPH was capable of inducing cell apoptosis and arresting the cell cycle in S phase. SPPH can also promote endogenous reactive oxygen species (ROS) generation and decrease mitochondrial membrane potential (MMP). Western blotting and transcriptome analysis demonstrated that SPPH treatment could affect the regulation of several important modulators and signalling pathways related to tumourigenesis, intrinsic apoptosis and cell cycle. Our results revealed the antitumour potential of SPPH.

## 2. Results

### 2.1. SPPH Preparation

Silkworm pupae were superfine crushed using disintegrator, and the crude protein of silkworm pupae was separated after degreasing treatment. After the processing of alkali dissolving and acid precipitating, the gross protein was produced. Then, alcalase was used to perform hydrolyzing of gross protein, and the degree of hydrolysis (DH) was increased with the extension of processing time (Figure S1). After treatment for 160 min, the DH reached to a steady state and almost completely degraded into small peptides and amino acids, which were validated by the hydrolysis curve (Figure S1). We defined the hydrolysis product as SPPH and used it to perform the following cytological and pharmacological analyses.

### 2.2. SPPH Specifically Inhibits SGC-7901 Cell Proliferation in a Dose- and Time-Dependent Manner

**Figure 1:**
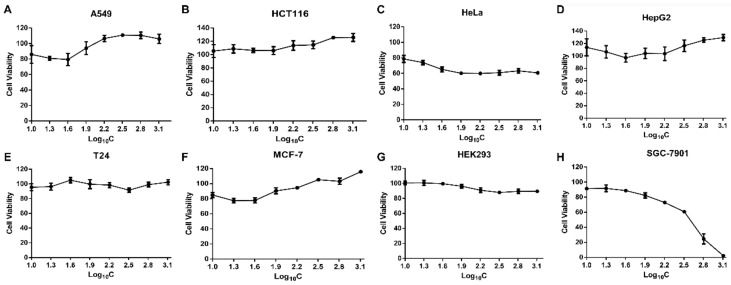
Cell proliferation evaluation by MTT assay under different concentrations of SPPH (silkworm pupa protein hydrolysate) treatment. (**A**–**H**) various human cell lines.

To assess the cytotoxicity of SPPH to different cancer cells that derived from different tumour tissues and HEK293 cell, we performed MTT assay with A549, HCT116, T24, Hela, SGC-7901, MCF-7, HepG2, and HEK293 cells. SPPH showed nearly no inhibiting effect on A549, HCT116, MCF-7, T24 and HepG2 cells and repressed the growth of HeLa cells with weak capability (Figure 1A–F). The proliferation rate of human embryonic kidney cells HEK293 was not affected by the SPPH, and their proliferative activity was similar to that of control after SPPH treatment (Figure 1G). However, the survival rate of SGC-7901 cells was decreased with the increasing of SPPH concentration and was almost zero when treatment concentration reached 1.28 mg/mL (Figure 1H). Moreover, we incubated SGC-7901 cells with SPPH for 12, 24 and 48 h and found that cell viability was decreased with the extension of treating time (Figure S2). This finding indicated that SPPH could specifically inhibit the growth of SGC-7901 cells with no side effect on normal cells.

**Figure 2:**
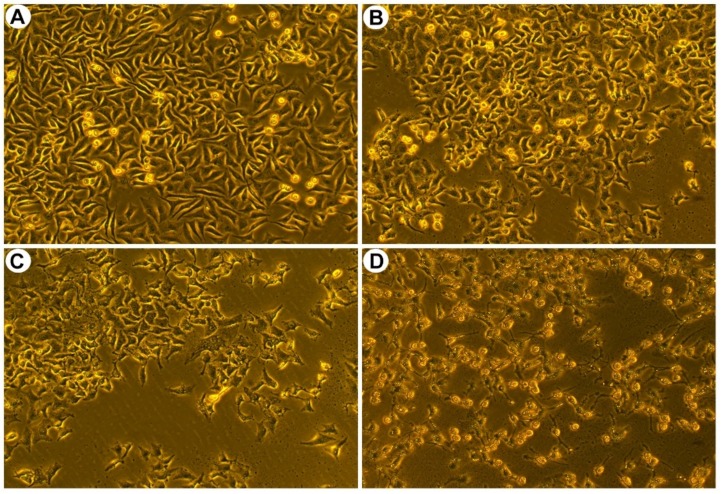
Morphological characteristics of SGC-7901 cells after treated with indicated concentrations of SPPH for 36 h. Morphological changes of SGC-7901 cells were observed after SPPH treated with different concentrations, (**A**) control; (**B**) 80 μg/mL; (**C**) 160 μg/mL; (**D**) 320 μg/mL.

Furthermore, morphological changes, such as cell shrinkage and cytoskeleton disintegration, are remarkable characters of apoptotic cells. To examine the changes of cell morphology after SPPH treatment, we performed morphological assay of SGC-7901 cells. Three SPPH concentrations, 80, 160 and 320 μg/mL, were selected to treat the cells, and the morphology of cells was observed under a phase contrast microscope. As a result, the non-treated cells were well-spread and flattened in cell plate, whereas SPPH-treated cells displayed apoptotic features with cell shrinkage and cytoplasmic condensation (Figure 2). Larger doses of SPPH led to the floating of massive SGC-7901 cells (Figure 2D). Therefore, SPPH specifically restrained the proliferation and induced apoptosis of SGC-7901 cells in dose- and time-dependent manner. Due to the selective and effective antitumour property of SPPH to SGC-7901 cells, we investigated the antitumour mechanism of SPPH to this gastric cancer cell.

### 2.3. SPPH Induces Apoptosis in SGC-7901 Cell

**Figure 3:**
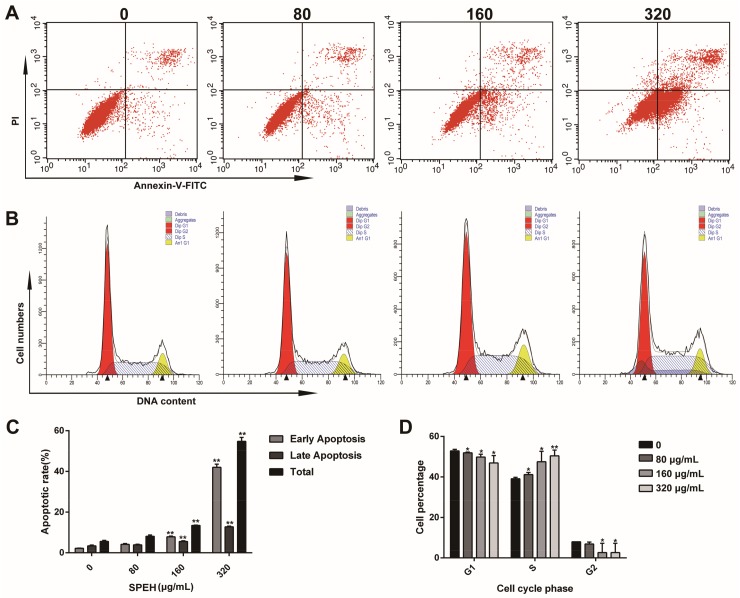
SPPH induces apoptosis and cell cycle arrest of SGC-7901 cells. (**A**) SGC-7901 cells were treated with SPPH for 36 h and labeled with Annexin V and PI to conduct flow cytometric assay; (**B**) SGC-7901 cells were stained with propidium iodide (PI) and subjected to flow cytometric assay; (**C**,**D**) Quantitative analysis for the cell apoptosis and cell cycle distributions, respectively. Data are expressed as Mean ± SD (*n* = 3). ** *p* < 0.01 versus control, * *p* < 0.05 versus control.

To further determine the apoptotic induction of SPPH to SGC-7901 cells, we performed the flow cytometry assay of SPPH-exposed SGC-7901 cells after Annexin V-FITC/PI staining. Compared with untreated cells, SPPH treatment significantly promoted apoptosis with increasing concentration of SPPH (Figure 3A,C). The percentages of early apoptosis and late apoptosis increased in a dose-dependent manner, from 2.23% to 42.00% and from 3.41% to 12.76%, respectively (Figure 3C). This finding indicated that SPPH induced the apoptosis of SGC-7901 cells by destroying the integrity of the plasma membrane.

### 2.4. SPPH Blocks the Cell Cycle of SGC-7901 Cell in S Phase

To evaluate the impact of SPPH treatment on SGC-7901 cell-cycle profile, we pretreated cells with SPPH in different concentrations and examined the distribution of cell cycle using flow cytometry. As a result, the cells in S phase significantly increased from 39.95% to 50.40% after SPPH exposure (320 μg/mL) for 36 h, whereas the cells in G_1_ phase remarkably decreased from 52.89% to 46.93% and cells in G_2_ decreased from 8.0% to 2.67% (Figure 3B,D). Therefore, SPPH can arrest the cell cycle of SGC-7901 cells at S phase in a dose-dependent manner.

### 2.5. SPPH Promotes ROS Generation of SGC-7901 Cell

**Figure 4:**
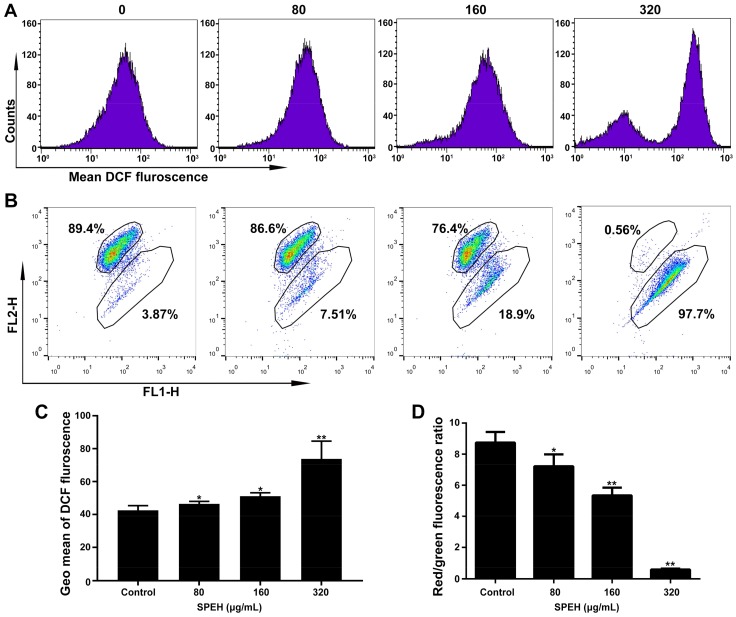
SPPH induced ROS generation and MMP loss in SGC-7901 cells. (**A**) After treatment with SPPH for 36 h, SGC-7901 cells were stained by ROS indicator (DCF-DA) and ROS levels were measured by flow cytometry; (**B**) MMP detection of SGC-7901 cell using JC-1 probe by flow cytometry; (**C**) Average DCF fluorescence, indicated by Geo Mean, was shown in the histogram; (**D**) Average fluorescence intensity was indicated by histograms. Data are expressed as Mean ± SD (*n* = 3). ** *p* < 0.01 versus control, * *p* < 0.05 versus control.

ROS activation has been reported to induce apoptosis in cancer cells [18,19]. We performed ROS detection using DCFA-DA fluorescent probe, which could be oxidized by ROS to produce fluorescent DCF. We found that treatment with SPPH resulted in highly increased fluorescence intensity compared with those of nontreated cells (Figure 4A). The value of Geo Means, an indication of fluorescence intensity, was increased from about 36 (control) to 73 (treatment with 320 μg/mL SPPH), suggesting a significant rising trend of ROS production when treated with SPPH (Figure 4C). SPPH treatment boosted ROS generation in a dose-dependent manner (Figure 4A,C). Moreover, ROS generation was reduced, and the viability of SPPH-treated cells was recovered after antioxidant NAC administration (Figure S3). This finding suggested that the antitumour effect of SPPH, at least partly, depends on promoting ROS generation.

### 2.6. SPPH Disrupts MMP of SGC-7901 Cell

Depolarization of MMP is a factor that contributes to cell apoptosis and may be accompanied by excessive ROS generation [20,21]. Therefore, we evaluated the MMP change of SGC-7901 after SPPH treatment using JC-1 staining. As shown in Figure 4B, cells with normal MMP were significantly decreased after treatment with 80, 160 and 320 μg/mL SPPH (89.4% to 86.6%, 76.4% and 0.56%, respectively). Moreover, relative quantification of MMP by Geo Means indicated that the red fluorescence was reduced, and the green fluorescence was remarkably increased after SPPH treatment (Figure 4D), indicating that MMP was disrupted after SPPH treatment.

### 2.7. SPPH Affects Protein Expression Related to Regulation of Intrinsic Apoptosis and Cell Cycle

**Figure 5:**
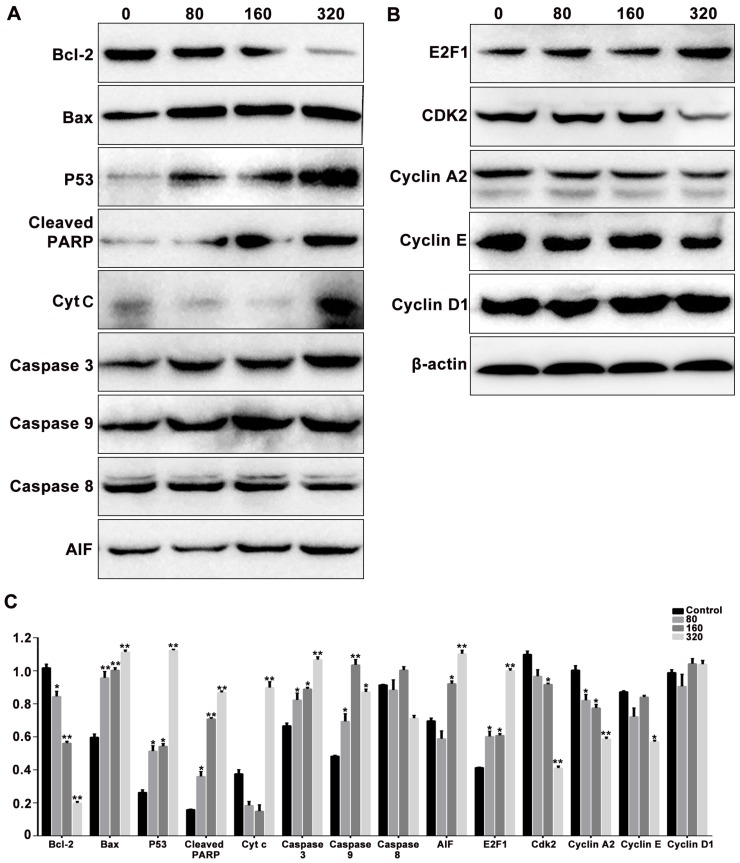
The effects of SPPH treatment on the expression levels of the apoptosis (**A**) and cell cycle (**B**) related proteins in SGC-7901 cells. Cells were treated with SPPH in different concentrations for 36 h, then applied to protein extraction and determination. β-actin was used as inner control. (**C**) Quantification of protein expression levels normalized to β-actin in the Western blotting analysis. Data are expressed as Mean ± SD of three independent experiments. * *p* < 0.05, ** *p* < 0.01 compared with the control.

**Figure 6:**
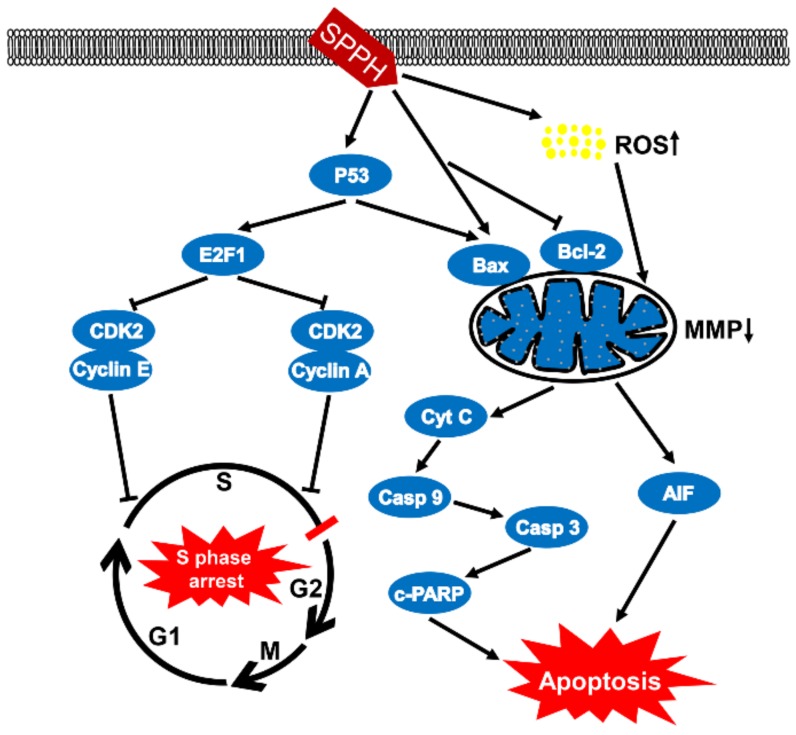
Proposed model for the mechanisms of SPPH-induced cytotoxicity in SGC-7901 cells. On the one hand, SPPH enhanced P53 expression to induce E2F1 and suppressed two complexes of CDK2 and Cyclin E, CDK2 and Cyclin A, which blocked the cell cycle in S phase. On the other hand, SPPH promoted ROS accumulation and induced the expression of Bax by enhancing the expression of P53, or induced Bax expression, inhibited Bcl-2 expression independently, destroyed MMP and induced Cyt C and AIF release to activate downstream caspase signal pathways and apoptosis. Casp 9, caspase-9; Casp 3, caspase-3; c-PARP, cleaved PARP.

Bcl-2 family members, Bcl-2 and Bax, can respond to apoptotic stimulus and can affect the homeostasis of mitochondria, then various apoptotic mediators can be activated to influence the series cascades of downstream involved in cell death [22]. To elevate the effects of SPPH treatment on the expression of apoptosis and cell-cycle regulators, we performed western blotting analysis of SGC-7901 cells. When treated with an increased dose of SPPH, the expression level of antiapoptotic regulator Bcl-2 was decreased, whereas the expression of proapoptotic protein Bax was increased (Figure 5A). Then, we examined the effects of SPPH on the expression level of other regulators in the intrinsic apoptotic pathway. As shown in Figure 5A, SPPH treatment facilitated mitochondrial release of cytochrome C (Cyt C) and mitochondrial flavoprotein apoptosis-inducing factor (AIF), activated the expression of initiator caspase-9 and executioner caspase-3, induced poly (ADP-ribose) polymerase (PARP) cleavage, eventually provoked intrinsic apoptosis and DNA fragmentation. However, the other initiator caspase regulator, caspase-8, was not significantly affected. Moreover, proapoptotic and antitumour protein P53 was activated after treatment with SPPH. Therefore, SPPH treatment could induce apoptosis by mediating the regulators of the intrinsic apoptotic pathway (Figure 6).

In addition, we determined the effects of SPPH treatment on the expression of cell-cycle regulators in SGC-7901 cells. As a result, SPPH treatment could activate the expression of E2F1 and could inhibit the expression of CDK2, Cyclin E and Cyclin A2 (Figure 5B). However, Cyclin D1 as a cell-cycle mediator not directly involved in S phase was not affected by SPPH. Therefore, S phase arrest induced by SPPH treatment was achieved by altering the expression of key governors in the cell-cycle regulation of S phase (Figure 6).

### 2.8. SPPH Affects Metabolic Regulation and Signal Transduction in Tumourigenesis of SGC-7901 Cells

**Figure 7:**
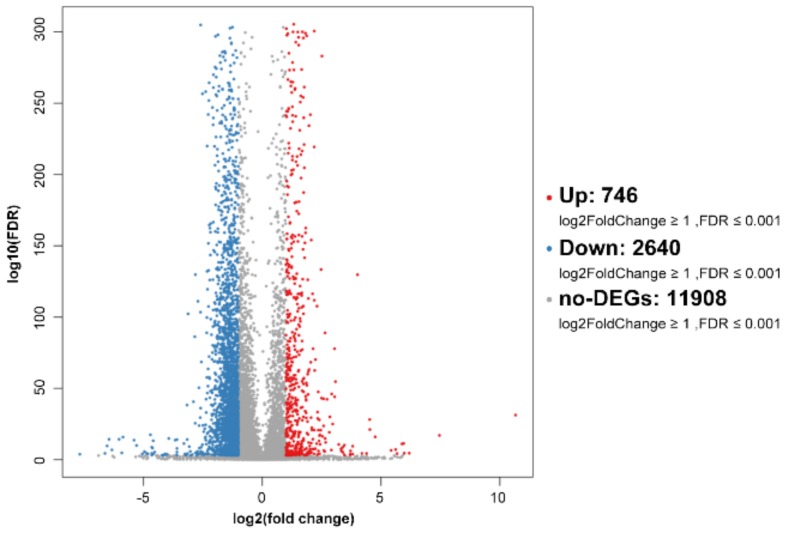
Volcano plot of global gene expression profile after SPPH treatment. Down- (blue dots) and up-regulated genes (orange dots) are presented as differentially expressed genes (DEGs).

To investigate the effect of SPPH on the gene expression of SGC-7901, we performed RNA-seq after SPPH treatment (320 μg/mL) using BGISEQ-500 platform. As a result, a total of 23.95 and 24.10 Mb clean reads were acquired after SPPH treatment, whereas 24.10 and 24.08 Mb clean reads were identified in the control group (Table S1). Then, the clean reads were mapped to Human genome (Hg19), and the total mapping ratios were 87.58%, 84.68% and 85.89%, 81.88% in treatment and control groups, respectively. A total of 746 up-regulated genes and 2640 down-regulated genes were identified after SPPH treatment using the filtering criteria: fold change ≥ 2.00 and FDR ≤ 0.001 (Figure 7). Among these genes, SPPH treatment could significantly induce the expression changes of 23 genes among annotated 74 genes directly involved in apoptosis, including coding genes for Bcl-2-related ovarian killer protein, Threonine-protein kinase LATS2, Mitofusin-2 and TNF receptor-associated factor 6 (Table 1). Moreover, SPPH treatment altered the expression of 40 genes in total 149 annotated genes directly involved in the cell cycle, including coding genes for CREB-binding protein, P300 protein, Transcription factor E2F3 and Cyclin-dependent kinase inhibitor 1B (Table 1). Many genes participated in the regulation of tumourigenesis were also affected by SPPH treatment (Table 1).

**Figure 8:**
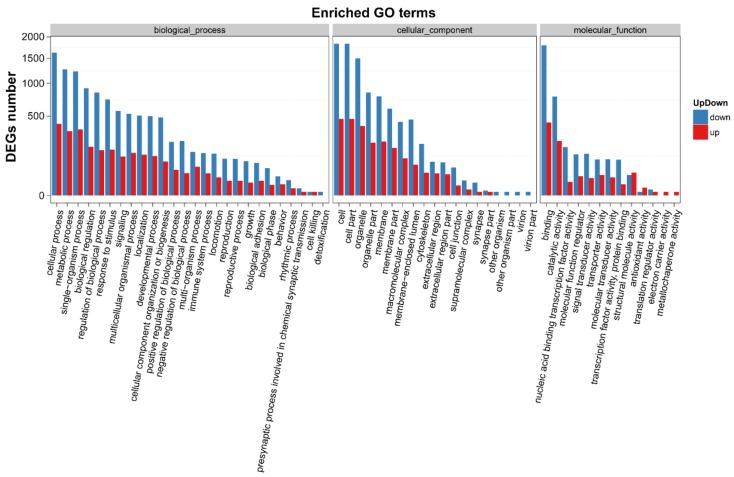
GO functional classifications of DEGs after SPPH treatment. The *x*-axis indicates the GO terms, and the *y*-axis indicates the number of DEGs. GO terms were grouped into three ontologies: biological process, cellular component and molecular function. Down- and up-regulated genes are presented in blue and red, respectively.

In addition, we performed gene ontology (GO) and Kyoto Encyclopedia of Genes and Genomes (KEGG) enrichment analyses to confirm the biological significance and functional classifications of the DEGs. GO analysis indicated that the filtered DEGs were involved in various biological functions when clustered into categories of molecular function, cellular component and biological process (Figure 8). In the molecular function category, cellular process (GO: 0009987), metabolic process (GO: 0008152) and single-organism process (GO: 0044763) were the top three largest subcategories that DEGs enriched in. In the category of cellular component, cells (GO: 0005623), cell parts (GO: 0044464) and organelle (GO: 0043226) were the major terms that DEGs located in. Under the category of biological process, DEGs were mainly grouped into binding (GO: 0005488), catalytic activity (GO: 0008152) and nucleic acid-binding transcription factor activity (GO: 0001071). 

**Figure 9:**
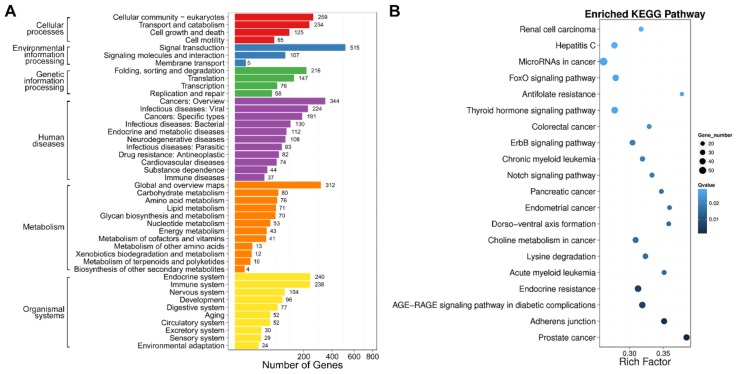
KEGG classification and enrichment of DEGs. (**A**) KEGG classification of DEGs. The *x*-axis indicates the number of DEGs, and the *y*-axis indicates the KEGG pathways; (**B**) Pathway enrichment of DEGs. Rich Factor indicates the ratio of differentially expressed gene numbers annotated for a pathway term relative to all the genes annotated with this pathway term. The *Q*-value is the corrected *p*-value ranging from 0 to 1. A higher Rich Factor and a lower *Q*-value represent greater intensity. Only the top 20 enriched pathway terms are displayed.

Subsequently, we mapped the DEGs to KEGG metabolic and regulatory pathways. Among these pathways, cellular-community eukaryotes, signal transduction, folding, sorting and degradation, cancers: overview/specific types, endocrine system and immune system were the main pathways that DEGs clustered into (Figure S4). Moreover, DEGs were enriched in a series of cancer-related pathways after SPPH treatment, including prostate cancer (ko05215), acute/chronic myeloid leukaemia (ko05221/ko05220), endometrial cancer (ko05213), pancreatic cancer (ko05212), colorectal cancer (ko05210), choline metabolism in cancer (ko05231) and microRNAs in cancer (ko05206) (Figure 9). Therefore, this finding demonstrated that SPPH treatment altered a wide range of regulatory pathways involved in tumourigenesis and cancer-related metabolic regulations.

GSEA was used to evaluate the gene sets that were differentially expressed between SPPH-treated SGC-7901 cells and the untreated cells. Our data indicated that SPPH treatment affected gene sets primarily associated with cell proliferation, cell adhesion, oxidative phosphorylation and mitochondrial structure and complex (Figure S5). Gene sets of cell proliferation and cell adhesion were positively enriched in the untreated cells; whereas gene sets of oxidative phosphorylation, mitochondrial membrane part, inner mitochondrial membrane protein complex and mitochondrial membrane protein complex were positively enriched in SPPH-treated cells (Figure S5). Thus, GSEA also suggested that SPPH treatment can suppress SGC-7901 cells growth by regulating cell proliferation, adhesion and mitochondrial structure and functions.

## 3. Discussion

Medical and health-care efficiencies of silkworm pupae have been detected since ancient times, such as reducing blood pressure and blood lipids, enhancing immunity, improving liver function and ameliorating malnutrition [2,5,6,23,24]. However, the antitumour activity of silkworm pupae has been rarely reported. Wu et al. found that the antibacterial peptides extracted from silkworm pupae could suppress S180 sarcoma growth and alleviate the ascites formation of Ehrlich Ascites Cancer [25]. However, the exact mechanisms involved remain mysterious and need more investigations. In this study, we revealed the antitumour effect of silkworm pupa compounds. SPPH could specifically inhibit the proliferation of SGC-7901 cells, and further assay demonstrated that this antitumour efficacy was induced by promoting cell apoptosis and arresting the cell cycle in S phase. Moreover, the expediting apoptosis function of SPPH was regulated by ROS-dependent intrinsic mitochondrial pathway. The antitumour property and mechanism of silkworm pupa compounds to human gastric cancer cell were first characterized in this study.

Apoptosis is an active and programmed cell death process, which is characterized by cell shrinkage and convolution, chromatin condensation and extensive plasma membrane blebbing [26]. Previous studies have uncovered two major pathways that regulate the apoptosis process, including mitochondria-mediated apoptotic intrinsic pathway and death receptor-induced extrinsic pathway. Deregulation of apoptosis leads to imbalance between cell proliferation and cell death, which may result in tumourigenesis. Therefore, restoring and promoting the apoptosis of cancer cells are convincing ways to treat cancers [27]. Other studies revealed that certain bioactive substances from natural products could exert antitumour effects through intrinsic apoptotic induction [28,29]. Here, SGC-7901 cells underwent apoptosis upon treatment with concentration-dependent SPPH supported by typical apoptotic features, including cell shrinkage, cytoplasmic condensation and phosphatidylserine translocation (Figure 2 and Figure 3A). Bcl-2 family plays a key role in the initiation of the intrinsic apoptotic pathway. In this study, SPPH was found to regulate the expression of Bcl-2 proteins by repressing the expression of Bcl-2 and inducing the expression of Bax (Figure 5A). Therefore, we hypothesized that the proapoptotic effect of SPPH was realized by activating the downstream regulators of intrinsic pathway. Western blotting data corroborated our speculation as the expressions of related intrinsic apoptotic regulators, including PARP, Caspase-3, Caspase-9, AIF and Cyt C changed significantly after SPPH treatment at the protein level. Moreover, transcriptome results revealed that SPPH treatment induced the expression changes in the genes directly involved in apoptosis, and the pathways that contributed to apoptosis were also fluctuated after SPPH treatment. GSEA supported that gene sets involved in cell proliferation and cell adhesion were positively enriched in untreated cells compared with SPPH-treated cells. In addition, p53 is a tumour suppressor, which mediates various genes with a wide range of functions, including apoptosis, cell-cycle regulation, DNA repair and senescence [30]. Here, SPPH treatment induced p53 expression in a dose-dependent manner, suggesting that SPPH treatment could suppress tumourigenesis and proapoptosis by provoking p53-dependent pathway.

Cell cycle is tightly regulated in healthy cells to ensure that specific events take place in an orderly manner [31,32]. Deregulation of tumour suppressors or activation of protooncogene could dismantle checkpoints that monitor progression through S/G2/M phases, increase the dependency of cancer cells on G1-phase cyclin-dependent kinases, as well as induce DNA damage and aggravate replication stress during S phase [33]. Corresponding therapies and drugs derived from natural products have been developed to target cancer cells with abnormal cell-cycle distribution [34,35,36]. In this study, we found that SPPH treatment was sufficient to arrest cell cycle in S phase (Figure 3B,D). E2F1 is a transcription factor that contributes to the regulation of the G1-to-S phase transition by interacting with cell-cycle regulators [37]. Here, SPPH induced the expression of E2F1, and E2F1 induction subsequently activated the expression of CDK2 and its binding partners Cyclin A and Cyclin E, which are two proteins involved in the regulation of the S phase of cell cycle and G1–S phase of the cell cycle, respectively (Figure 5B). Therefore, the S phase arresting effect of SPPH was dependent on the activation of key regulators in S phase progression (Figure 6). The transcriptome analysis also provided lines of evidence that SPPH treatment could modify cell-cycle control in SGC-7901 cells (Table 1). Moreover, activated p53 has been proven to regulate the binding of P21 to CDK2 and induce cell-cycle arrest [38]. Therefore, the cell-cycle-arresting effect by SPPH treatment should also be related to the upregulation of P53.

ROS is mainly produced by mitochondria in cells, and its overproduction has been proven to destroy cell microenvironments, and even leads to cell death [39]. Previous studies revealed that ROS accumulation could bring about mitochondrial dysfunction by depolarizing the MMP, and triggering mitochondria-mediated proapoptotic signals [18,40]. Consequently, MMP disruption initiates the release of AIF and Cyt C to activate downstream apoptotic signals, such as Apaf-1, SMAC and caspases [41,42]. Our study revealed that SPPH treatment induced ROS accumulation in SGC-7901 cells and caused a dose-dependent depolarization of MMP and upregulation of AIF and Cyt C (Figure 4 and Figure 5A). Therefore, SPPH could promote ROS accumulation to activate the mitochondria-mediated apoptotic intrinsic pathway. The GSEA results indicated that this effect may be raised by the regulations of oxidative phosphorylation and structure changes of mitochondrial complex. We also speculated that SPPH treatment could provoke impaired antioxidant capacity in the mitochondria of SGC-7901 cells. This antitumour mechanism of SPPH treatment, by regulating ROS generation, is similar to those of the amino acids from selenium-rich silkworm pupae to human hepatoma cell SMMC-7721 [43]. Furthermore, previous studies have demonstrated that the excessive production of intracellular ROS and mitochondrial dysfunction could prevent cell-cycle progression, such as blocking the cell cycle in S phase [44,45]. In our study, SPPH was sufficient to block the cell cycle in S phase, and this effect could be partly caused by accumulating intracellular ROS level in this cancer cell. For these reasons, the SPPH-induced ROS accumulation deeply influenced various aspects of SGC-7901 cells and is a complex factor for the antitumour effect of SPPH treatment. However, whether SPPH-induced proapoptosis is involved in other signals and pathways, such as death receptor-induced extrinsic pathway and endoplasmic reticulum stress signals, is an opening question remains to be addressed.

Taken together, this study indicated that SPPH specially and significantly suppressed SGC-7901 cell proliferation by promoting intrinsic apoptosis and blocking the cell cycle in S phase. This apoptosis-induction effect was closely related to intracellular ROS levels and in a mitochondria-dependent manner. In addition, further work is needed to resolve the precise composition of SPPH that exerts antitumour effects on SGC-7901 cells. Nowadays, the requirement of antitumour drugs with excellent safety and efficiency is urgent as certain anticancer drugs in clinical application can elicit harmful side effects on normal cells [46]. Our results suggest a possibility to use peptides extracted from silkworm pupae for the specific therapeutic management of gastric cancer in the future.

## 4. Materials and Methods

### 4.1. Materials

Silkworm (*Bombyx mori*, strain: Qiufeng × Baiyu) pupae were reared in our laboratory. Alcalase (2.4 L) was purchased from Novozymes (Copenhagen, Denmark). 3-[4,5-Dimethylthiazol-2-yl]-2,5-diphenyl-tetrazolium bromide (MTT) and dimethyl sulfoxide (DMSO) were purchased from Sigma-Aldrich (St. Louis, MO, USA). Dulbecco’s modified Eagle’s medium (DMEM) and 0.25% Trypsin (with 0.5% EDTA) were purchased from HyClone (Logan, UT, USA). Fetal bovine serum (FBS) was purchased from Gibco (Grand Island, NY, USA). Annexin V-FITC/PI Apoptosis Detection Kit was purchased from Sungene Biotech (Tianjin, China). Cell Cycle Detection Kit was purchased from KeyGEN Biotech (Nanjing, China). Antioxidant NAC, Reactive Oxygen Species Assay Kit and Mitochondrial Membrane Potential Assay Kit (JC-1) were purchased from Beyotime Institute of Biotechnology (Haimen, China). All other chemicals were obtained from commercial sources of analytical grade.

For western blotting analysis, the primary antibodies against AIF, BCL-2 and Bax were purchased from HUABIO Biotechnology (Hangzhou, China). The primary antibodies against CDK2, Caspase-3, Caspase-9, Cyt C, E2F1, Cleaved-PARP, P53, E2F1, CDK2, cyclin A2, cyclin D1 and cyclin E were purchased from Cell Signaling Technology (Beverly, MA, USA). Horseradish peroxidase (HRP)-conjugated secondary antibody was purchased from Abcam (Cambridge, MA, USA). One-Step Animal Cell Active Protein Extraction Kit was purchased from Sangon Biotech (Shanghai, China). BCA Protein Assay Kit was purchased from Pierce (Rockford, IL, USA). Immobilon Western Chemiluminescent HRP Substrate was purchased from Millipore (Atlanta, GA, USA). Materials and chemicals for electrophoresis were from Bio-Rad (Hercules, CA, USA).

### 4.2. Cell Lines and Cell Culture

The human lung adenocarcinoma cell A549, human colon cancer cell HCT116, human breast cancer cell MCF-7, gastric cancer cell SGC-7901 and human bladder carcinoma cell T24 were provided by the Cell Bank of Type Culture Collection of Chinese Academy of Sciences (Shanghai, China). The human cervical carcinoma cell Hela, human hepatoma cell HepG2 and human embryonic kidney cell HEK293 are preservative cell line of our Lab. All cells were maintained in DMEM medium supplemented with 10% fetal bovine serum under 5% CO_2_ at 37 °C in a humidified atmosphere. The cells were digested and passaged by 0.25% trypsin-EDTA when reached 80% confluence.

### 4.3. Isolation and Extraction of SPPH

Fresh silkworm pupae were heat-treated at 120 °C and then smashed by high-speed pulverizer. The chopped samples were degreased thrice with petroleum ether (1:3) for 24 h. The defatted silkworm chrysalis was heat-treated at 50 °C to remove the remaining petroleum ether. The uniform defatted pupa powder was obtained after filtration through 80-mesh filter. The defatted pupa powder was dissolved in distilled water (1:20), and the pH was adjusted to 9 with 5% NaOH solution. After stirring for 1 h, the solution was centrifuged at 3000× *g* for 30 min, and the supernatant was collected. The above supernatant was dissolved, and the pH was adjusted to 4 with HCl. After centrifuging at 3000× *g* for 10 min, the pupa protein was obtained in precipitates and freeze-dried using LABCONCO Freeze Dryer (Kansas City, MO, USA).

After dissolving in distilled water at 10 mg/mL, the pupa protein was hydrolyzed by Alcalase at 50.8 °C, and the pH was maintained at 9 for 4 h through stirring. The enzyme amount is 3500 U/g for hydrolysis. Then, the enzymatic reaction was terminated by placing in boiling water for 10 min and cooled it to room temperature. After centrifuging at 5000× *g* for 10 min, the supernatant was collected and freeze-dried for storage in a dryer.

### 4.4. Cell Viability Assay

To assess the cytotoxicity of SPPH, we selected the above 7 cancer cells derived from different tumour tissues and HEK293 cell to analysis the cell viability after SPPH treatment. The cell viability assay was performed using the MTT method. Target cells, which were cultured to logarithmic growth phase, were seeded in 96-well plates with 6 × 10^3^ cells/well. After culturing for 24 h, the SPPH of varied concentrations were added to wells, and the cells were processed for 24–48 h. After dropping the supernatant, 10% MTT solution was added to each well and incubated for 4 h. 150 μL DMSO was added to wells and oscillated with low speed for 10 min to dissolve the formazan crystals. The absorbance measurement was conducted at 490 nm by a BioTek microplate reader (Winooski, VT, USA). The ratio of cell viability was calculated as follows:

Cell viability (%) = (A_490 sample_ − A_490 blank_)/(A_490 control_ − A_490 blank_) × 100. Moreover, the IC_50_ values were determined by using the GraphPad Prism 6 (GraphPad, San Diego, CA, USA).

### 4.5. Cell Apoptosis Examination

To assess the morphological changes of SGC-7901 cells after SPPH treatment, SGC-7901 cells were seeded in 6-well plate and incubated in gradient concentrations of SPPH for 36 h. The cells were fixed with fixative solution overnight and then observed under an Olympus phase contrast microscope.

SGC-7901 cell apoptosis examination was investigated using Annexin V-FITC/PI Apoptosis Detection Kit according to the manufacturer’s instruction. The cells in logarithmic growth phase were collected and dispersed in 6-well plates with 2 × 10^5^ cells/well. Different concentrations of SPPH were added to wells and incubated for 36 h. After washing thrice with ice-cold PBS, treated cells were collected and resuspended in 300 μL binding buffer. Annexin V-FITC (5 μL) was added to the suspension and reacted for 10 min in dark, and PI (5 μL) was added and reacted for 15 min in dark. Flow cytometry analysis was performed by BD FACSCalibur (Becton Dickinson, San Jose, CA, USA).

### 4.6. Cell-Cycle Determination

SGC-7901 cells in logarithmic growth phase were collected and dispersed in 6-well plates with 2 × 10^5^ cells/well. Different concentrations of SPPH was used to treat cells and then incubated for 36 h. The cells were collected and fixed with 70% ethanol overnight. After washing twice with ice-cold PBS, the cells were resuspended in 100 μL RNase buffer for 30 min at 37 °C. Then, 400 μL PI-staining solution was added to the suspension and reacted for 30 min at 4 °C under dark condition. Flow cytometry analysis was performed by BD FACSCalibur (Becton Dickinson, San Jose, CA, USA) and Modfit LT software 3.1 (Verity Software House, Topsham, ME, USA).

### 4.7. Measurement of Endogenous ROS Generation

ROS Assay Kit was used to determine the ROS generation of SGC-7901 cells, which was indicated by 2′,7′-dichlorofluorescein-diacetate (DCFH-DA) staining. The cells were harvested and treated with SPPH in different concentrations for 36 h. Pretreated DCFH-DA (10 μmol/L) was used to incubate cells at 37 °C for 20 min. After washing the cells thrice, fluorescence intensity, an indicator of relative changes at intracellular ROS level, was determined by BD FACSCalibur (Becton Dickinson, San Jose, CA, USA).

### 4.8. MMP Assay

MMP assay kit with JC-1 probe was used to investigate the MMP changes after SPPH treatment. After treated by SPPH in different concentrations for 36 h, SGC-7901 cells were incubated with JC-1 (20 μg/mL) at 37 °C for 20 min and then washed twice with JC-1 staining buffer. Treated cells were observed by a fluorescent microscope, and the flow cytometry analysis was conducted using BD FACSCalibur (Becton Dickinson, San Jose, CA, USA).

### 4.9. Western Blotting Assay

After SPPH treatment for 36 h, SGC-7901 cells were harvested and washed with cold PBS twice. The total protein was extracted by using One Step Animal Cell Active Protein Extraction Kit (Sangon Biotech, Shanghai, China) according to the manufacturer’s instructions. Extracted protein concentration was determined by using the BCA Protein Assay Kit (Pierce, Rockford, IL, USA). Equivalent amounts of protein samples (20 μg) were separated by 12% sodium dodecylsulfate polyacrylamide gel electrophoresis (SDS-PAGE) and then transferred to PVDF membranes (Millipore, Atlanta, GA, USA). After being blocked in blocking buffer (5% nonfat dry milk/0.05% Tween 20 in 20 mM TBS at pH 7.4) at room temperature for 2 h, the membranes were incubated with primary antibodies for 2 h at room temperature, followed by incubating with horseradish peroxidase conjugate (HRP)-conjugated secondary antibodies at room temperature for 1 h. The signals were detected by Immobilon Western Chemiluminescent HRP Substrate (Millipore, Atlanta, GA, USA).

### 4.10. RNA Extraction, cDNA Library Construction and Sequencing

SGC-7901 cells were treated with SPPH for 36 h, collected, washed thrice with ice-cold PBS, and stored at −80 °C before extraction. RNA extraction was performed using Trizol Reagent (Invitrogen, Life Technologies, Carlsbad, CA, USA). Untreated SGC-7901 cells under normal growing conditions were used as a control. The 23S and 16S rRNA were depleted by MicrobExpress Kit (Ambion, Austin, TX, USA). Genomic DNA was removed by using Amplification-grade DNase 1 (Invitrogen, Life Technologies, Carlsbad, CA, USA). RNA integrity was verified using Agilent 2100 Bioanalyzer with Agilent RNA 6000 Nano Kit (Agilent Technologies, Waldbronn, Germany). Then, the RNA was sheared and reverse transcribed with random primers to obtain cDNA to conduct library construction. The quality of constructed library was measured using Agilent 2100 Bioanalyzer. Qualified libraries were subsequently sequenced using BGISEQ-500 platform at BGI (Shenzhen, China). Two biological repetitions of SPPH treatment and control were performed in RNA-seq. The transcriptome raw sequencing datasets are available from Sequence Read Archive database in NCBI, and their accession number is SRP128982.

### 4.11. Data Analysis of RNA-Seq

Raw reads acquired by sequencing were filtered to remove the adaptor reads, reads with unknown bases more than 10% and low-quality reads (the ratio of the bases with quality value *Q* ≤ 15 greater than 50%) using the SOAP nuke software (available online: http://soap.genomics.org.cn/). Clean reads were mapped to reference genome using HISAT software (available online: http://www.ccb.jhu.edu/software/hisat). Bowtie2 (available online: http://bowtie-bio.sourceforge.net/Bowtie2/index.shtml) was used to map the clean reads to the reference genes, and the gene-expression level was quantified by RSEM with default parameters (available online: http://deweylab.biostat.wisc.edu/RSEM). PossionDis was used to determine significantly differentially expressed genes (DEGs) after SPPH treatment (fold change ≥2.00 and false discovery rate (FDR) ≤0.001).

The Gene ontology (GO) functional enrichment and Kyoto Encyclopaedia of Genes and Genomes (KEGG) pathway analyses of DEGs were performed by hypergeometric distribution testing using the Phyper function of the R software package (available online: http://www.r-project.org/). FDR correction was used to determine the threshold *p*-value in multiple tests, and FDR ≤ 0.01 was regarded as a significant enrichment.

The Gene set enrichment analysis (GSEA) was performed using the Broad Institute’s GSEA tool (available online: http://www.broadinstitute.org/gsea/index.jsp) to gain further insight into the pathway gene sets that were correlated with SPPH treatment. Default parameters were used. The gene sets were obtained from the molecular signature database of the Broad Institute (available online: http://software.broadinstitute.org/gsea/msigdb/index.jsp).

### 4.12. Statistical Analysis

All experiments were conducted at least three times. Statistical analyses were conducted using GraphPad Prism 6 (GraphPad, San Diego, CA, USA). Data were represented as mean ± SD or as indicated. One-way ANOVA was used to compare the values of experimental and control groups. Student’s *t*-test was employed to confirm the significance when only two groups were compared. Differences were considered statistically significant or extremely significant when *p* < 0.05 or <0.01, respectively.

## Figures and Tables

**Table 1:** Changes in the transcriptional expression of key genes related to cell apoptosis, cell cycle and tumorigenesis in response to SPPH treatment. Fold-changes are indicated for each gene significantly up- or down-regulated (*p* < 0.05, fold-change > 2) in SPEH-treatment group compared with the control.

Gene ID	Description	*p* Value	Regulated	Fold-Change	
				Control	SPEH-Treated
**Apoptosis**					
666	Bcl-2-related ovarian killer protein	2.80 × 10^−157^	Up	17.49	45.73
706	Translocator protein	0	Up	203.29	704.69
26524	Threonine-protein kinase LATS2	5.36 × 10^−95^	Down	7.47	1.79
142	Poly (ADP-ribose) polymerase 1	0	Down	78.43	20.94
143	Poly (ADP-ribose) polymerase 4	2.38 × 10^−60^	Down	7.8	2.85
7189	TNF receptor-associated factor 6	1.04 × 10^−15^	Down	1.22	0.43
673	Threonine-protein kinase B-raf	9.78 × 10^−9^	Down	1.79	0.65
9927	Mitofusin-2	0	Down	55.68	20.37
60485	Protein salvador homolog 1	3.89 × 10^−56^	Down	15.2	6.2
2000	ETS-related transcription factor Elf-4	6.88 × 10^−93^	Down	21.43	9.19
**Cell cycle**					
10459	Mitotic spindle assembly checkpoint protein MAD2B	0	Up	99.57	257.9
51529	Anaphase-promoting complex subunit 11	1.20 × 10^−84^	Up	43.2	95.73
1387	CREB-binding protein isoform b	4.00 × 10^−307^	Down	10.11	1.68
2033	P300 protein	3.46 × 10^−244^	Down	10.86	2.25
4172	DNA replication licensing factor MCM3	0	Down	111.26	40.41
8243	Structural maintenance of chromosomes protein 1A	0	Down	22.26	5.74
9134	G1/S-specific cyclin-E2	7.65 × 10^−20^	Down	7.89	3.71
1871	Transcription factor E2F3			11.63	3.88
23126	Pogo transposable element with ZNF domain	3.46 × 10^−93^	Down	9.49	3.19
1030	Cyclin-dependent kinase 4 inhibitor B	3.12 × 10^−79^	Down	14.19	5.06
1027	Cyclin-dependent kinase inhibitor 1B	3.23 × 10^−111^	Down	36.99	14.84
**Tumorigenesis**					
6923	Transcription elongation factor B polypeptide 2 isoform a	0	Up	207.92	777.89
5881	Ras-related C3 botulinum toxin substrate 3	0	Up	52.68	190.19
5595	Mitogen-activated protein kinase 3	9.47 × 10^−133^	Up	35.33	76.26
112399	Egl nine homolog 3 isoform 2	1.49 × 10^−35^	Up	3.82	10.69
140775	Guanine nucleotide exchange protein SMCR8	2.48 × 10^−121^	Down	5.56	1.13
8817	Fibroblast growth factor 18	4.17 × 10^−15^	Down	2.81	0.62
284217	Laminin subunit α-1 precursor	2.38 × 10^−48^	Down	2.1	0.49
2263	Fibroblast growth factor receptor 2	9.33 × 10^−107^	Down	11.76	2.82
4790	Nuclear factor NF-κ-B p105 subunit	3.07 × 10^−53^	Down	6.15	1.57
7473	Proto-oncogene Wnt-3	6.06 × 10^−15^	Down	2.59	0.87

## Supplementary Materials

Supplementary materials can be found at http://www.mdpi.com/1422-0067/19/4/1013/s1.

## Abbreviations

SPPH	silkworm pupa protein hydrolysate
DH	degree of hydrolysis
AIF	apoptosis-inducing factor
Cyt C	cytochrome C
MMP	mitochondrial membrane potential
ROS	reactive oxygen species
CDK	cyclin-dependent kinase
PARP	Poly (ADP-ribose) polymerase
PBS	phosphate-buffered saline
HRP	horseradish peroxidase
FBS	fetal bovine serum
MTT	3-[4,5-Dimethylthiazol-2-yl]-2,5-diphenyl-tetrazolium bromide
PI	propidium iodide
FITC	fluorescein isothiocyanate
DMSO	dimethyl sulfoxide
DMEM	Dulbecco’s modified Eagle’s medium
DCFH-DA	2′,7′-dichlorofluorescein-diacetate
BCA	bicinchoninic acid
ACE	angiotensin-I-converting enzyme
FDR	false discovery rate
DEGs	differentially expressed genes
KEGG	Kyoto Encyclopedia of Genes and Genomes
GO	gene ontology
GSEA	gene set enrichment analysis
