# Supplementary material for: Silkworm Pupa Protein Hydrolysate Induces Mitochondria-Dependent Apoptosis and S Phase Cell Cycle Arrest in Human Gastric Cancer SGC-7901 Cells
Source: Int J Mol Sci. 2018 Mar 28;19(4):1013. doi: 10.3390/ijms19041013 (PMC5979490; doi:10.3390/ijms19041013)
Supplement: Supplementary file 1 [file ijms-19-01013-s001.pdf]

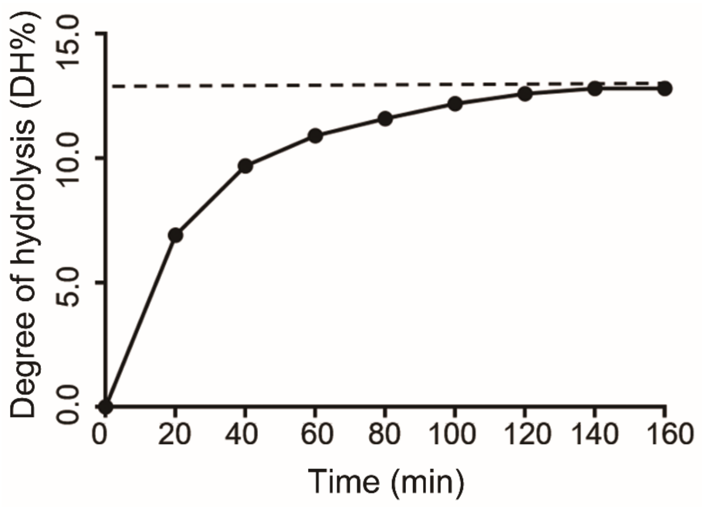

Figure S1. The hydrolysis curve of silkworm pupae by alcalase.

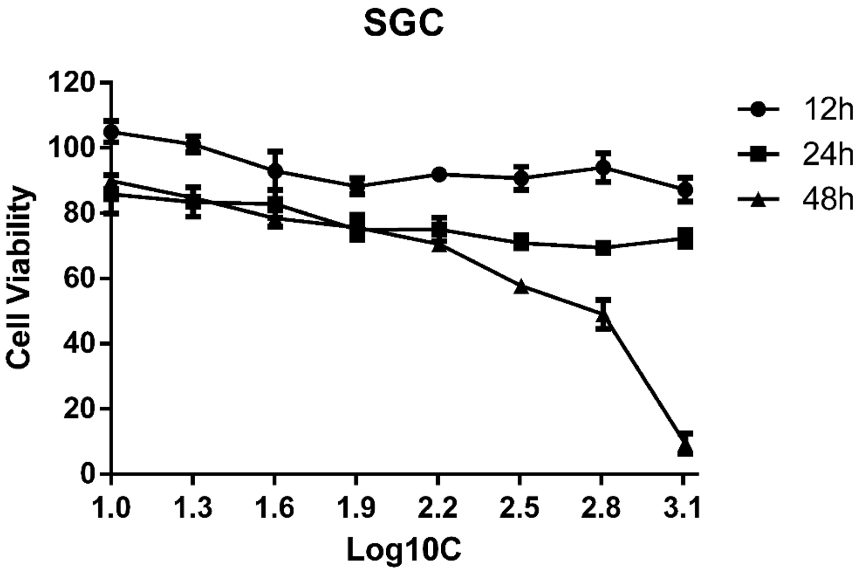

Figure S2. SPPH can inhibit SGC-7901 cell proliferation in a time-dependent manner.

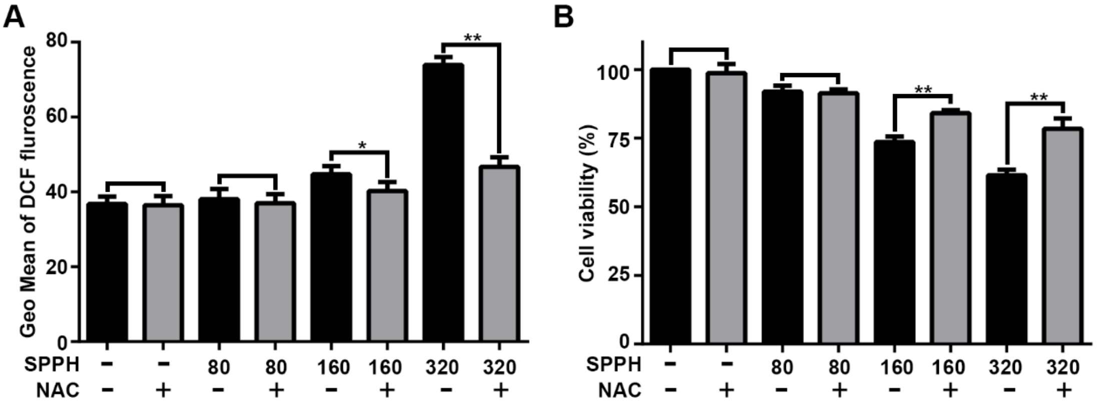

Figure S3. NAC (5mM) treatment can repress the SPPH-mediated ROS generation (A) and recover SPPH-mediated cell death (B). ROS generation was indicated by ROS indicator (DCF-DA) and ROS level was measured by flow cytometry. Cell viability was measured by using MTT method. SPPH treated with different concentrations 80, 160 320 μg/mL. Data are expressed as Mean ± SD (n=3). ** $P<0.01$ versus control, * $P<0.05$ versus control.

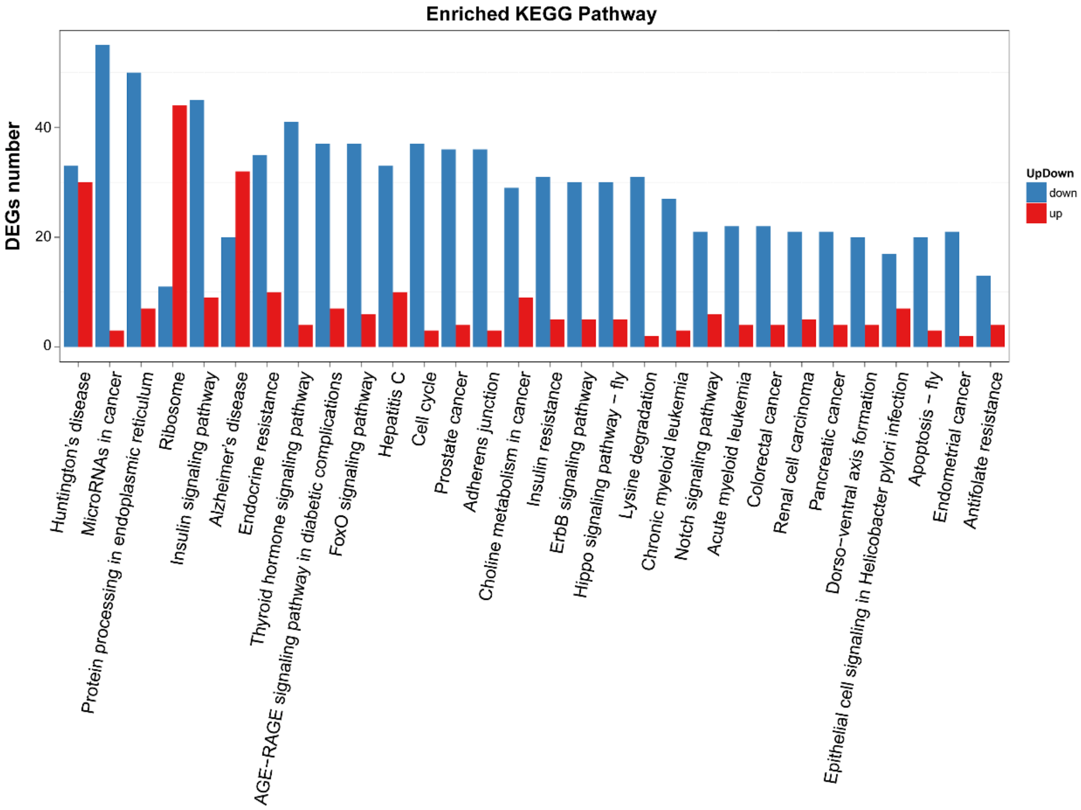

Figure S4. KEGG classification of DEGs.

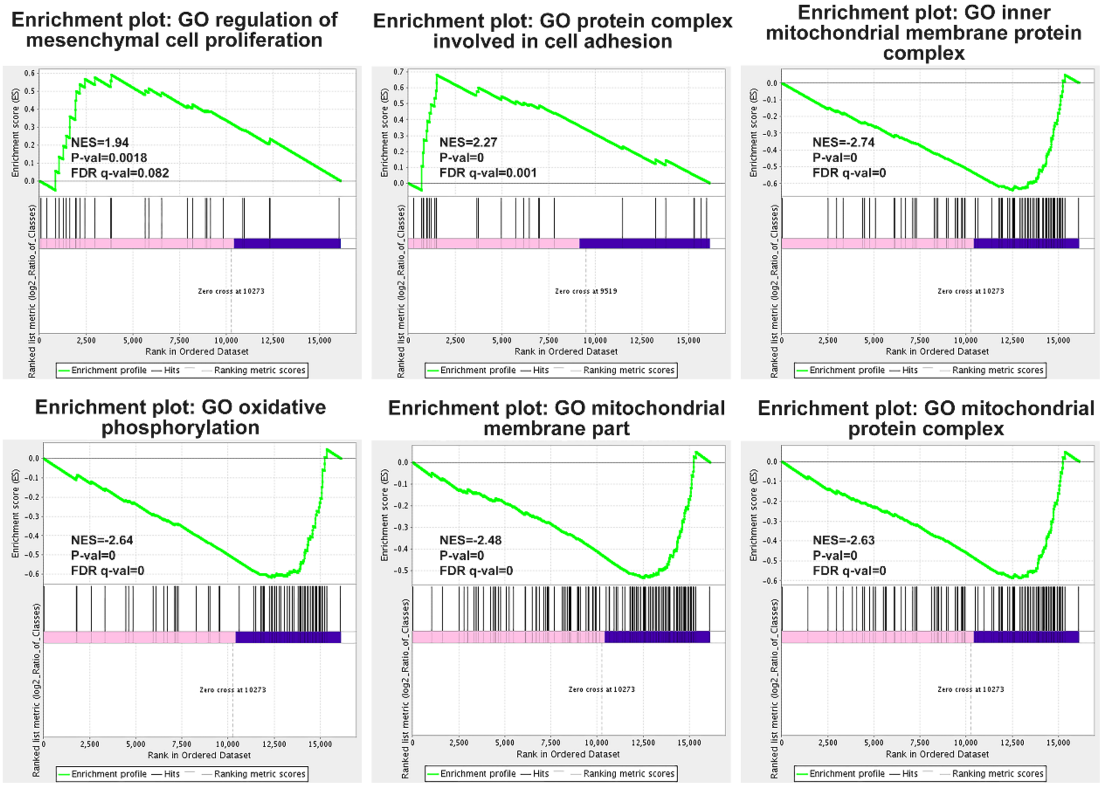

Figure S5. GSEA revealed key enriched pathways after SPPH treatment.

Table S1. Summary of transcriptome sequencing, assembly and annotation

Sample	Total Raw Reads (Mb)	Total Clean Reads (Mb)	Total Clean Bases (Gb)	Clean Reads Q20 (%)	Clean Reads Q30 (%)	Clean Reads Ratio (%)	Total Mapping Ratio (%)	Unique Match (%)
Control-1	24.14	24.08	1.20	98.25	91.14	99.74	81.88	76.25
Control-2	24.14	24.10	1.20	97.17	88.61	99.84	85.89	80.93
SPEH-treated-1	24.14	23.95	1.20	98.39	91.53	99.22	87.58	81.38
SPEH-treated-2	24.14	24.10	1.21	97.21	88.77	99.85	84.68	79.84
